# Environmental public health research at the U.S. Environmental Protection Agency: A blueprint for exposure science in a connected world

**DOI:** 10.1038/s41370-024-00720-8

**Published:** 2024-11-16

**Authors:** Lindsay W. Stanek, Wayne E. Cascio, Timothy M. Barzyk, Michael S. Breen, Nicole M. DeLuca, Shannon M. Griffin, Lisa Jo Melnyk, Jeffrey M. Minucci, Kent W. Thomas, Nicolle S. Tulve, Christopher P. Weaver, Elaine A. Cohen Hubal

**Affiliations:** 1 U.S. Environmental Protection Agency, Office of Research and Development, Center for Public Health and Environmental Assessment, Research Triangle Park, NC 27707 USA; 2 U.S. Environmental Protection Agency, Office of Research and Development, Center for Public Health and Environmental Assessment, Cincinnati, OH 45268 USA; 3 Present Address: Research Triangle Institute International, Research Triangle Park, NC 27709 USA

**Keywords:** Climate change, Cumulative impacts, Disease, Exposomics, Non-chemical, Stressors

## Abstract

**Abstract:**

Exposure science plays an essential role in the U.S. Environmental Protection Agency’s (U.S. EPA) mission to protect human health and the environment. The U.S. EPA’s Center for Public Health and Environmental Assessment (CPHEA) within the Office of Research and Development (ORD) provides the exposure science needed to characterize the multifaceted relationships between people and their surroundings in support of national, regional, local and individual-level actions. Furthermore, exposure science research must position its enterprise to tackle the most pressing public health challenges in an ever-changing environment. These challenges include understanding and confronting complex human disease etiologies, disparities in the social environment, and system-level changes in the physical environment. Solutions will sustainably balance and optimize the health of people, animals, and ecosystems. Our objectives for this paper are to review the role of CPHEA exposure science research in various recent decision-making contexts, to present current challenges facing U.S. EPA and the larger exposure science field, and to provide illustrative case examples where CPHEA exposure science is demonstrating the latest methodologies at the intersection of these two motivations. This blueprint provides a foundation for applying exposomic tools and approaches to holistically understand real-world exposures so optimal environmental public health protective actions can be realized within the broader context of a One Health framework.

**Impact statement:**

- The U.S. EPA’s Center for Public Health and Environmental Assessment exposure research priorities reside at the intersection of environmental decision contexts and broad public health challenges. The blueprint provides a foundation for advancing the tools and approaches to holistically understand real-world exposures so optimal environmental protection actions can be realized. A One Health lens can help shape exposure research for maximum impact to support solutions that are transdisciplinary and must engage multiple sectors.

## Introduction

Exposure science plays an essential role in the U.S. Environmental Protection Agency’s (U.S. EPA) mission to protect human health and the environment. Research to understand how people are exposed to environmental stressors is critical for providing the foundation for credible decision-making to safeguard human health and ecological integrity. Exposure science, for the purposes of this paper, is the study of human contact with chemical, physical, biological, or psychosocial stressors in the lived environment [1–4]. Exposure assessment is a key step in the traditional source-to-outcome framework connecting the external environment and internal dose [1]. Historically, human health risk assessments have been conducted for a single stressor in a single medium. This simplified approach has proved valuable for informing policies, rules and regulations that reduce or eliminate exposure to harmful chemicals and pathogens. Though exposures to chemical mixtures and multiple stressors have been evaluated, challenges persist in operationalizing methodologies to incorporate this information for decision-making [5]. It has long been recognized that exposure science is at the nexus of complex interactions between humans and the environment, and exposures need to be investigated holistically to address cumulative impacts of human activities and conditions [4, 6]. Recent recommendations from the National Academies of Science, Engineering and Medicine (NASEM) encourage U.S. EPA to implement systems thinking in concert with a One Environment-One Health (One Health) approach “…to enhance ORD’s scientific capability for considering the complex interactions among environmental, social, and economic systems…” [7].

The U.S. EPA’s Center for Public Health and Environmental Assessment (CPHEA) within the Office of Research and Development (ORD) provides the science needed to characterize the interrelationships between people and nature in support of Agency and stakeholder assessments and policy decisions to protect human health and the environment [8]. As the home of U.S. EPA public health science, CPHEA is uniquely situated to conduct research under the One Health construct, “a collaborative, multisectoral, and transdisciplinary approach — working at the local, regional, national, and global levels — with the goal of achieving optimal, [sustainable], health outcomes…” [9]. The purpose of this commentary is to describe the necessary advancements in exposure science to support public health challenges now and in the coming years. This blueprint recognizes the need to conduct specific applied exposure science research to fulfill the U.S. EPA’s mission, while also considering the broader real-world context. We provide case examples of how CPHEA is currently achieving both objectives, while also preparing for the multifaceted exposure science challenges of the future using the latest tools to optimize public health decisions.

## Exposure science decision contexts

Exposure science informs decisions across a range of stakeholders and scales. CPHEA’s research demonstrates the cross-cutting nature of exposure science, reflecting applications across public, private, government, and academic stakeholders at geographic scales ranging from individuals and communities, to states, multi-state regions, and nationwide. Our research outputs address environmental health issues of national importance, while also recognizing that these impacts are happening to real people in the real world, in the communities where they live, learn, work, and play. In this respect, CPHEA research further emphasizes the importance of place in a person’s overall exposure, illustrating that many environmental issues are common across the country, yet every community is unique in its combinations of exposures and the actions that could mitigate them.

The following selected case examples from recent exposure science research conducted by CPHEA speak to these national- and local-scale decision-making contexts.

### National scale for regulatory decisions

Exposure science research is a key responsibility of ORD, as the U.S. EPA was created with a research arm to provide the scientific basis for regulatory policies and to support the emerging needs of Agency stakeholders, including state, territorial, tribal, and community partners [8]. This differs substantially from Agencies such as the National Institutes of Health and National Science Foundation where the mission is to fund and conduct research that advances fundamental knowledge [7]. These agencies provide valuable scientific contributions to advance societal well-being, although the relevance and applicability of the science to U.S. EPA policy-making can be limited. The outputs of CPHEA’s exposure research directly inform national rules developed by the U.S. EPA’s land, water, air, and chemical Program Offices. Recent research activities have focused on extending traditional chemical and pathway specific exposure assessments to address more complex human-environment systems.

#### Exposure modeling for Pb

Lead (Pb) is a ubiquitous chemical found in many different environmental media, including drinking water, house dust, soil and air. Therefore, understanding the contributions from different exposure pathways is critical to better determine how to reduce exposure and blood lead levels (BLL) and mitigate the long-term adverse health effects of Pb toxicity. CPHEA researchers combined a stochastic predictive exposure model with a Pb deterministic biokinetic and uptake model to estimate aggregate exposures and BLL and contributions to BLL from multimedia exposure pathways [10]. The advanced modeling system was evaluated using national scale blood Pb data from the CDC’s National Health and Nutrition Examination Survey (NHANES) and showed agreement. This modeling approach was used by the U.S. EPA’s Office of Water to support the Lead and Copper Rule [10, 11]. The relative source contribution analyses demonstrated the importance of water ingestion for infants and soil/dust ingestion for toddlers in predicting BLL.

A critical input to Agency exposure assessments is inadvertent soil and dust ingestion; this variable has been shown to be highly sensitive in predictions of children’s BLL [10]. CPHEA researchers have led recent modeling efforts to apply the Stochastic Human Exposure and Dose Simulation model for Soil and Dust (SHEDS-Soil/Dust model) to derive new exposure scenarios for infants, predict soil/dust ingestion rates for different groups of adults, and estimate separate soil and dust ingestion rates [12–14]. Another possible approach is to pinpoint a novel tracer using non-targeted laboratory techniques that could be used to calculate children’s dust ingestion rates, but this is yet to be fully realized; a workflow has been proposed to identify and rank organic chemical candidates [15].

#### Per- and polyfluoroalkyl substances (PFAS) occurrence in exposure media

PFAS are a broad group of compounds that have unique properties which have resulted in widespread use and considerable environmental contamination [16]. Exposure to impacted communities through contaminated drinking water is a health concern. However, the presence of PFAS chemicals in 98% of NHANES samples suggests other exposure sources and pathways [17, 18]. CPHEA researchers and collaborators are investigating important pathways of exposure to PFAS by applying a data mining approach to the existing literature for measured occurrence of PFAS in exposure media [19–21]. The U.S. EPA’s Office of Water used this information to support selection of the Relative Source Contribution (the allocation of exposure attributable to nondrinking water sources) in setting drinking water health advisory levels for several PFAS. In addition, secondary data from exposure and health studies are being analyzed to understand variation in exposure sources and archived specimens are being analyzed to further build the evidence base and inform decisions that minimize and mitigate exposures in the residential environment [22].

#### Occupational exposure to airborne organic chemicals

Occupational exposure to hazardous chemicals is estimated to cause over 290,000 deaths each year, and it represents a key piece of an individual’s exposure because working-age adults spend a large proportion of time at their place of work [23]. Additionally, the chemicals present in work environments differ dramatically from those in the home and may be highly job specific. For the U.S. EPA to effectively regulate existing and emerging chemicals under the 2016 Frank Lautenberg Chemical Safety for the 21st Century Act, tools must be developed to screen chemicals for job-specific occupational exposure risks. To address these challenges, CPHEA and other ORD researchers led the effort to leverage over 30 years of Occupational Safety and Health Administration workplace air monitoring data to train a Bayesian model that predicts detection frequency and air concentration for organic chemicals [24]. This model used a hierarchical workplace classification system and the predicted physicochemical properties of the substance to derive estimates. Predicted air concentrations from this model can be used as inputs to exposure models to estimate worker doses and could be combined with high-throughput exposure estimates for other pathways, such as ambient air, consumer product and dietary sources, to build a more complete picture of individual exposures.

### State and local scale for community decisions

CPHEA works closely with the Agency’s Regional Offices to collaborate with stakeholders, including state, territorial, tribal, and community partners, to address pressing public and environmental health issues and inform a range of actions. External partners often have flexibility to maneuver multiple decision-making levers to affect change to prevent or mitigate exposure to chemical and biological agents at the community and individual scale. It is imperative that we work with these groups proactively as a component of early research planning to identify the decision context and then optimize data collection to obtain the information that is most relevant. This teamwork principle is consistent with One Health, where establishing partnerships so all perspectives can be recognized is critical to successful study implementation and results dissemination [7]. An important distinction is that for national policy makers and the broader research community, peer reviewed scientific journal articles are the currency for making results broadly available; for communities who need actionable science to bring about positive change in their environment, other tools are required to deliver results. The subsections that follow highlight tangible case studies when CPHEA scientists applied this practical approach to meet the needs of local partners.

#### Southwest Rockford Revitalization Rapid Health Impact Assessment (HIA)

The City of Rockford, Illinois, is revitalizing a de-industrialized neighborhood known as the South Main Corridor, which has a history of manufacturing and is now populated by low-income residents with low English proficiency. CPHEA researchers and partners from the Agency’s Office of Land and Emergency Management worked with city planners to identify possible health impacts of revitalization and offer strategies that reflect the priorities and values of the city planners and community residents and aimed to maximize positive impacts and minimize negative ones. The final HIA provided information on the most influential decisions, such as housing and commercial development, while also addressing the needs of the community, such as preserving cultural values and concerns over gentrification [25]. Over 80 strategies across social, environmental, and economic sectors were included in the HIA to maximize positive impacts of redevelopment. For example, while part of a larger effort, this HIA is helping inform city planners on the redevelopment of the Barber-Colman manufacturing complex, an abandoned 22-acre industrial complex that is now being renovated into 964 living units and 130,000 square feet of commercial space with an investment of over $400 million.

#### Assessment of tribal consumption of anadromous fish in Maine

Native American tribes have unique traditional cultural practices that differ from other communities. Recent river restoration efforts near the reservation of the Penobscot Indian Nation in Maine, including dam removals, have resulted in the abundant return of several anadromous fish species, where they have been missing for the past 200+ years. These returning fish potentially represent the restoration of a major component of the traditional diet, but contaminant levels in these fish were unknown. CPHEA researchers, working with ATSDR colleagues, characterized contaminant levels (e.g., mercury, dioxins, furans, PCBs, PBDEs and PFAS) in these fish that were compared to reference doses (where possible) and to previously published wildlife values [26, 27]. Using the contaminant results, a culturally appropriate risk assessment was developed to determine the potential risk Penobscot Tribal citizens face when engaging in their legally protected right of sustenance fishing and their traditional cultural practices [28]. The information was used to update tribal fish advisories [29].

### Personal decisions

There is also a need to translate and incorporate environmental exposure science into educational products and programs for allied health professionals, and into information and guidance accessible to individuals that can motivate and empower health protective behaviors. This is considered a growth area for CPHEA and efforts are underway for translating research results for greater utility by the general public.

## Environmental public health challenges

**Fig. 1:**
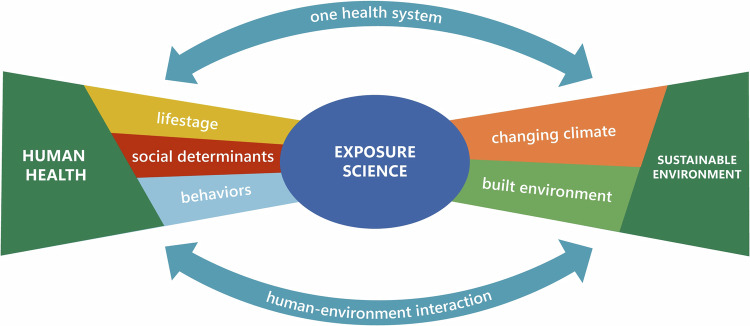
Environmental public health challenges being addressed by CPHEA exposure science research. Exposure science lies at the center of human health and sustainable environment. Key determinants of human health include lifestage, social determinants, and behaviors; key determinants of sustainable environment include changing climate and the built environment. A systems approach recognizing the human-environment interaction, through a One Health framework, provides an approach for consideration of cumulative impacts and the interconnection between people, animals, plants, and their shared environment.

With recent advances in scientific capabilities and knowledge yielding greater understanding of the multifaceted nature of challenges currently facing society, CPHEA is tackling the most pressing environmental public health problems that are within the scope of U.S. EPA’s mission (Fig. 1). By identifying and characterizing common exposure sources and pathways for multiple stressors and linking them to meaningful health outcomes, CPHEA can develop actionable strategies and solutions. While we break out the largest challenges currently being faced into separate categories below, they are all interconnected in a complex milieu in the real world.

### Tackling complex human diseases

Noncommunicable diseases have the highest disease burden worldwide, estimated to cause 74% of global deaths and 47% of global disability-adjusted life years in 2019 [30]. Diseases such as cardiovascular disease, metabolic syndrome, developmental disabilities, and cancer manifest through the complex interplay among behavioral and environmental influences, genetics, and pathophysiological responses [31–34]. The impact from exposures to chemical and non-chemical (e.g., biological and physical) stressors on health depends on timing. Exposures that occur during critical periods of development may result in health effects to the developing organism as well as manifest during later life stages [35–38]. Progress has been made in the environmental health sciences to investigate chemical exposures that individuals encounter, and the factors that contribute to onset of these chronic diseases [39].

Human activities and behaviors are at the core of exposure, where they inform health risk and can then be targeted for reduction or prevention efforts. It has been estimated that 50–90% of cardiovascular disease is modifiable through a healthy lifestyle [31, 40]. Lifestage and the corresponding activities are key individual-level exposure determinants for complex diseases and are well demonstrated for childhood outcomes [35, 41]. The complexity of health outcomes due to exposures to multiple environmental stressors must be considered in the broader context of physical and social environments [42, 43].

The Exposome refers to all the environmental exposures that an individual experiences over their lifetime where environmental exposure is broadly defined to include diet, activities, experiences, and the locations where we live, learn, work and play [39, 44, 45]. As methodologies and approaches for measuring the cumulative influence of environment on health over the life course advance, exposure modeling will enable comprehensive evaluation of relationships between stressors from the built, natural, and social environments [46, 47].

### Addressing disparities in the social environment

In addition to individual-level determinants, social conditions at the neighborhood and community level have a major influence on human exposure and disease etiology [48, 49]. Factors including economic stability, access to quality services (e.g., food, education, health care), safe housing and transportation, and social cohesion, to name a few, are considered social determinants of health [4, 50–52]. The inequitable distribution within the population of non-chemical stressors on well-being contributes to disparities in health outcomes. For instance, living in socially deprived neighborhoods increases coronary heart disease risk, even when adjusting for individual socioeconomic position [53, 54].

While social determinants of health accurately encapsulate our scientific understanding of many of the place-based influences on health outcomes, they are realized through disproportionate exposures to chemical and non-chemical stressors. Analogously, we can characterize this as social determinants of exposure. The inequitable distribution of environmental hazards that originate from historical social and political decisions (e.g., redlining) leading to marginalized communities, are part of the exposure context and should be considered as part of cumulative impacts and the exposome [55–57]. Probing underlying experiences of racism, social stress and marginalization is necessary to develop targeted and effective public health interventions [58].

### Confronting challenges in the physical environment

Human activities over the past century have changed the environment, and the impacts are substantial and far reaching. The interconnectedness of our world as demonstrated through economic, geopolitical, social, and technical advancements has enabled great progress and societal benefits and also unintended consequences [59].

Climate change is being felt worldwide, through alterations in precipitation patterns and temperatures, rising sea levels, wildfires, and increasing extreme weather events [60, 61]. These conditions produce additional individual and community-level stressors, and they can increase the occasions for human interaction with a variety of environmental agents that are normally controlled or prevented [62, 63]. For instance, flooding can disrupt drinking water and wastewater distribution systems or contaminated sites that may result in unique exposure scenarios to multiple biological, physical, and chemical stressors. In the U.S., socially vulnerable groups may be more likely to shoulder the burden of exposures to changing climate. For example, Black and African American, Hispanic and Latino, American Indian and Alaska Native, and Asian individuals are 20% more likely to live in areas that are projected to have global warming or coastal flooding [64].

Over the past several years, the increasing size and severity of wildland fires is well documented, substantially the result of human-driven climate change and longstanding fire suppression policies, and is projected to continue for decades into the future [65]. Harmful smoke from these episodes can impact humans and wildlife many miles removed from the fire itself. While these large fires have become commonplace in the western United States, recent episodes have also impacted the North Central, Northeast, and Mid-Atlantic regions. Additional factors are also increasing the number of people at-risk from wildfire smoke, including the expansion of the wildland-urban interface and an aging U.S. population. While the obvious impacts of poor air quality on public health have been shown, wildland fires can also affect water quality, vectors of disease and possibly the transportation of infectious fungal spores [66–69].

Over the past twenty years, sustainable building and urban design has emerged as a priority for governments to reduce environmental impacts and enhance benefits to occupants and the surrounding community [70]. Environmentally responsible development maximizes efficiency and creates positive physical, psychological, and social health [71]. The nine foundations of a healthy building include ventilation, air quality, thermal health, moisture, dust and pests, safety and security, water quality, noise, and lighting and views [71]. With North Americans spending an average of nearly 90% of their time indoors and an additional 6% in an enclosed vehicle, ample occasion exists for direct and indirect contact with chemical and biological sources and reservoirs [72, 73]. A wide variety of chemicals from building materials, furnishings, consumer products and other household items can migrate into air or dust becoming a source of exposure [20, 74, 75]. Similarly, biological materials and allergens are regularly found indoors. The presence and magnitude of chemicals and microbes inside structures are driven by environmental and ventilation factors, as well as human activity patterns [76, 77]. With a changing climate, mechanical operations of buildings (e.g., homes, schools, offices, commercial spaces) are changing to meet needs of their occupants, resulting in exposures that differ from those in past years. A more comprehensive understanding of indoor exposure in the context of broader societal and systematic settings in which exposures occur is needed [78].

## A blueprint for the future

**Fig. 2:**
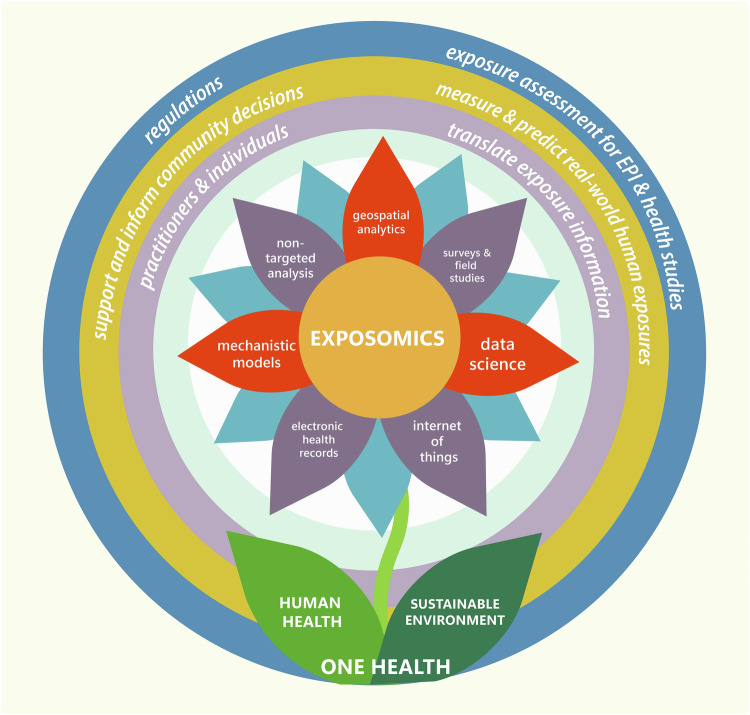
Blueprint for exposure science applications in CPHEA. CPHEA exposure research priorities reside at the intersection of environmental decision contexts (outer circles) and broad public health challenges (leaves). Examples of exposomic approaches (petals) provide the ways that the research is accomplished; some of these areas require partnering with external organizations (purple shading). A One Health lens can help shape exposure research for maximum impact. EPI = epidemiology.

CPHEA’s exposure research priorities reside at the intersection of environmental decision contexts and broad public health challenges. To be successful, we need to grow our exposure science research to enable timely decisions and actions that promote human health and a sustainable environment within a One Health system. Our approach is to access exposomic information and tools to: characterize the scope and magnitude of the most important environmental health problems; develop new information to fill the most critical knowledge gaps; and advance methods and tools to inform proactive public policy, private sector practices, and consumer behavior (Fig. 2). We must simultaneously be ready to adapt as the exposure environment continues to change rapidly, in response to climate change and other population forces. Advancement of holistic techniques to comprehensively characterize and quantify cumulative exposures, including all relevant environmental factors that disproportionately impact underserved and understudied populations, is a key area of ongoing research.

We continue to build capacity to apply state-of-the-science methodologies including non-targeted analysis and suspect screening of biological and environmental samples, internet of things (IOT; defined as a network of devices embedded with sensors, software and technology that facilitates communication) data collection using wearable and remote sensing technologies, and extant big data including electronic health records and geospatial information. This requires interdisciplinary collaboration among researchers of varying technical skills, including chemistry, engineering, statistics, biology, and social science. CPHEA’s exposure science leaders are bringing these critical disciplines together to use the latest approaches to answer the challenges ahead (Table 1). Both mechanistic (physically and biologically informed) modeling as well as data science techniques will be used to glean insights and predict potential for decisions and actions to affect change.

**Table 1:** CPHEA blueprint examples.

Example	Decision-Context	Challenge(s) being Addressed	Tools being Applied
Residential PFAS	National, regional, local scales	Physical environment	NTA, geospatial analytics, data science, surveys and field studies
Child health outcomes	National, regional, local scales	Complex disease, disparities in social environment	Surveys and field studies, data science
Waterborne disease	Regional, local scale	Complex disease, disparities in social environment, physical environment	Surveys and field studies, geospatial analytics
Personal exposure	Individual scale	Complex disease, disparities in social environment, physical environment	Surveys and field studies, geospatial analytics, mechanistic models, data science
ABM	National, regional, local scales	Disparities in social environment, physical environment	Internet of things, mechanistic models, geospatial analytics, data science

Research has focused on elucidating human exposure sources and pathways for contaminants and agents in the built environment that may be ubiquitous or emerging. This becomes even more difficult to disentangle when a class of chemicals that behave similarly are considered, such as perfluorinated compounds. Current exposure science research in CPHEA is utilizing non-targeted analysis of serum, soil, and bulk dust to identify emerging PFAS compounds in and around residences of communities impacted by aqueous film forming foam as well as those without known contamination of their drinking water. Additional data science approaches are combining information curated from the literature, aggregated from national sources, generated in the laboratory from available house dust and serum specimens, and collected by supplementing existing cohort studies to develop exposure-based categories for PFAS based on sources and occurrence patterns [21, 79–81]. Place-based data are serving as the basis for machine learning models that spatially predict where PFAS exposures may be greatest and further sampling should occur [82].

Understanding how non-chemical stressors interact with chemical stressors within the context of multiple public health challenges are areas of active research by CPHEA exposure scientists and epidemiologists. An ongoing effort is taking advantage of data from the National Children’s Study Vanguard Studies to examine the interrelationships between chemical exposures and a combined measure of social vulnerability based on social determinants of health (SDoH) as identified in the Protocol for Responding to and Assessing Patient Assets, Risks, and Experiences (PRAPARE) for a cohort of pregnant women in the U.S [83, 84]. The goal is to explore analyses of the combined interrelationships between chemical exposures, SDoH-based vulnerabilities, and child health outcomes in the NCS Vanguard Study cohort. By exploring clustering and other approaches for combining related non-chemical stressors, data reduction techniques can quantify relationships for cumulative impact assessments, where exposure information forms the foundational construct. In addition, CPHEA scientists have performed a systematic literature review for chemical and non-chemical stressors associated with childhood obesity and are currently building on this effort in a new community-based participatory research effort in Philadelphia, PA to better understand how the total environment, in combination with lived experience, cumulatively impacts this important multi-factorial health outcome [85].

CPHEA exposure scientists are working closely with communities affected by climate change who may be disproportionately impacted by environmental exposures to pathogens. We are investigating the link between community health and drinking water treatment in rural Puerto Rico. Through utilizing an innovative salivary fluid biomarker assay developed in-house, we are at the forefront of translational efforts to provide data critical to identifying, managing, and mitigating risks associated with waterborne disease in a vulnerable population [86–88].

In this era of personalized medicine that is focused on individual biology and risk factors, personalized exposure must be a component of CPHEA’s exposure research portfolio. Providing exposure data to individuals that is understandable and actionable is consistent with personalized medicine and is one direction we are pursuing. For example, CPHEA researchers are exploring the development of a publicly available smartphone application using data obtained from wearable sensors and low-cost air pollution monitors, which derives individual-level exposure in real-time [89]. We are currently leveraging the latest available sensor technology to incorporate additional exposure metrics (e.g., greenspace, noise, temperature).

Agent-based models (ABM) allows for a better understanding of exposure across time and space by considering an individual’s social and demographic factors, behavior, and local environment [51]. Exposure estimates can be improved by using ABM to estimate an individual’s behavior, movement through the environment, and interaction with other individuals based on time-activity pattern data and understanding of mediating factors. ABM simulate the behaviors and interactions of autonomous agents within a system, allowing us to model these patterns at scale of individuals and explore how behavior, demography, and social factors drive exposure to harmful chemicals or stressors [90, 91]. Continued development of ABM for human exposure will allow our exposure science to better capture demographic, social and behavioral differences in exposure and better address issues of environmental justice.

Together, these strategic capabilities will advance CPHEA exposure science that enables One Health solutions. Recent research conducted to support prioritization of sampling for PFAS contamination in the Columbia River Basin (CRB) in the northwestern United States serves as an example. A collaborative approach was applied to engage stakeholders from the CRB Restoration Working Group to identify exposures of concern [92]. Because the CRB is home to communities whose traditions, livelihoods and diet depend on local fish and wildlife, a more holistic need was identified to consider fish as sentinel species to predict sources of PFAS in the environment with impacts on both wildlife and human health.

CPHEA researchers used the limited existing fish tissue PFAS occurrence data available in the CRB, as well as publicly available geospatial data, to develop predictive models that could help decision-makers identify potential hotspots of PFAS contamination in fish tissue in the CRB [82]. Mapped predictions for Washington and Oregon showed several areas that had not been previously investigated where there may be potential for PFAS contamination in fish tissue; a recent study reported measured concentrations of PFAS in fillet samples from the region consistent with predictions [93]. Additionally, non-chemical stressors were analyzed to give insights about potential community-level vulnerability to PFAS exposure within the CRB [82]. The modeling approach and insights from this analysis recognize the interconnection between local communities, wildlife, and their shared environment. These data-driven and geospatial approaches to modeling PFAS hotspots in environmental media and identifying drivers of non-chemical stressors in communities provides a pilot example within the One Health context for decision-makers in the CRB to fill gaps in PFAS exposure characterization and better focus their efforts to reduce human exposure in impacted communities.

## Conclusions

Building upon ORD’s 35-year history of investigating cumulative exposures, CPHEA is well positioned to tackle exposure science research for the next generation of environmental decisions [6, 94–96]. As we expand our understanding into the complexity of the exposome and all its possible permutations, CPHEA seeks to ground its work in the decisions that the Agency and its environmental health stakeholders regularly face. To achieve these goals, collaboration with external partners is essential. In addition to building internal capacity in the latest techniques, we will continue to prioritize collaborations with other federal and state agencies, as well as tribal nations, academics, and NGOs to develop exposure information to best serve the purposes of our partners. By leveraging outside talent and assets of experienced partners, we will be able to efficiently advance the exposure science required by the U.S. EPA to meet increased pace and scale of health and environmental challenges [7]. Furthermore, with a renewed focus on solutions-driven research, partnerships with experts in science communication will be enhanced so that resulting scientific insights can be accessed and used by practitioners and communities.

Individual level differences in behavior, environment, genotype, and lived experience all contribute to human exposure and response, and ultimately, public health. CPHEA is poised to incorporate understanding of time, space, and circumstance when and where exposures occur through the latest exposomic methodologies to holistically address the nation’s most pressing problems, whether through national-level support for U.S. EPA’s regulations, state and local scale decision-making, or through empowering individual choice.
